# Supplementary material for: Modeling the impact of drug interactions on therapeutic selectivity
Source: Nat Commun. 2018 Aug 27;9:3452. doi: 10.1038/s41467-018-05954-3 (PMC6110842; doi:10.1038/s41467-018-05954-3)
Supplement: Supplementary file 1 — Supplementary Information [file 41467_2018_5954_MOESM1_ESM.pdf]

Supplementary Information

Modeling the impact of drug interactions on therapeutic selectivity

Weinstein et al

Supplementary Figure 1: Selectivity due to single agent variation in inhibitory concentration levels.

Supplementary Figure 2: Flow diagram of selectivity analysis.

Supplementary Figure 3: Drug combinations have a greater range of selectivity than single agents.

Supplementary Figure 4: Selectivity metric with respect to drug interaction scores (α).

Supplementary Figure 5: Selectivity metrics with respect to delta-α drug interaction scores by level of resistance.

Supplementary Figure 6: Validation of the selectivity model using flow cytometry.

Supplementary Figure 7: Evaluation of fungicidal activity of 12 compounds.

Supplementary Figure 8: Comparison of MMS-Rap selectivity score by growth metrics.

Supplementary Figure 9: Colony counts of MMS-Rap co-culture assay.

Supplementary Figure 10: Comparison of growth metrics.

Supplementary Figure 11: Comparison of interaction score by growth metrics.

Supplementary Figure 12: Observed selectivity by angle of checkerboard assay.

Supplementary Figure 13: Comparison of selectivity score by growth metrics.

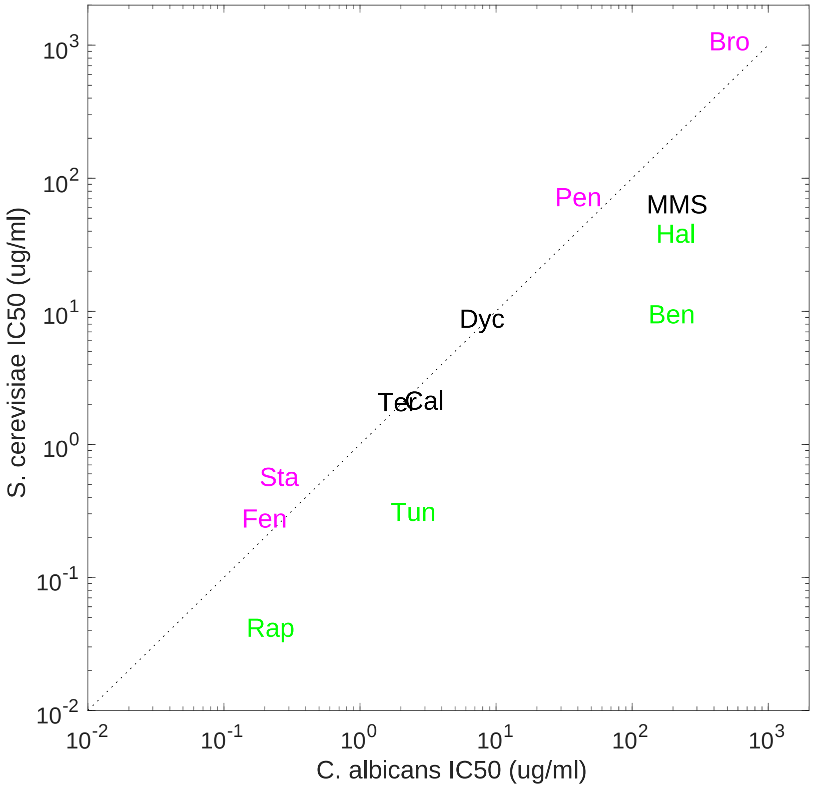

Supplementary Figure 1: Selectivity due to single agent variation in inhibitory concentration levels.

A comparison of the concentration required to inhibit the growth of *C. albicans* and *S. cerevisiae* by 50% (IC50). There was a strong positive correlation between IC50 levels of these yeast species ($r = 0.91$, $p < 10^{-13}$). We defined drugs as selective if there was a two-fold difference in IC50. Despite the high correlation, some drugs were less effective against one of the species (*C. albicans*-selective, green; *S. cerevisiae*-selective, magenta).

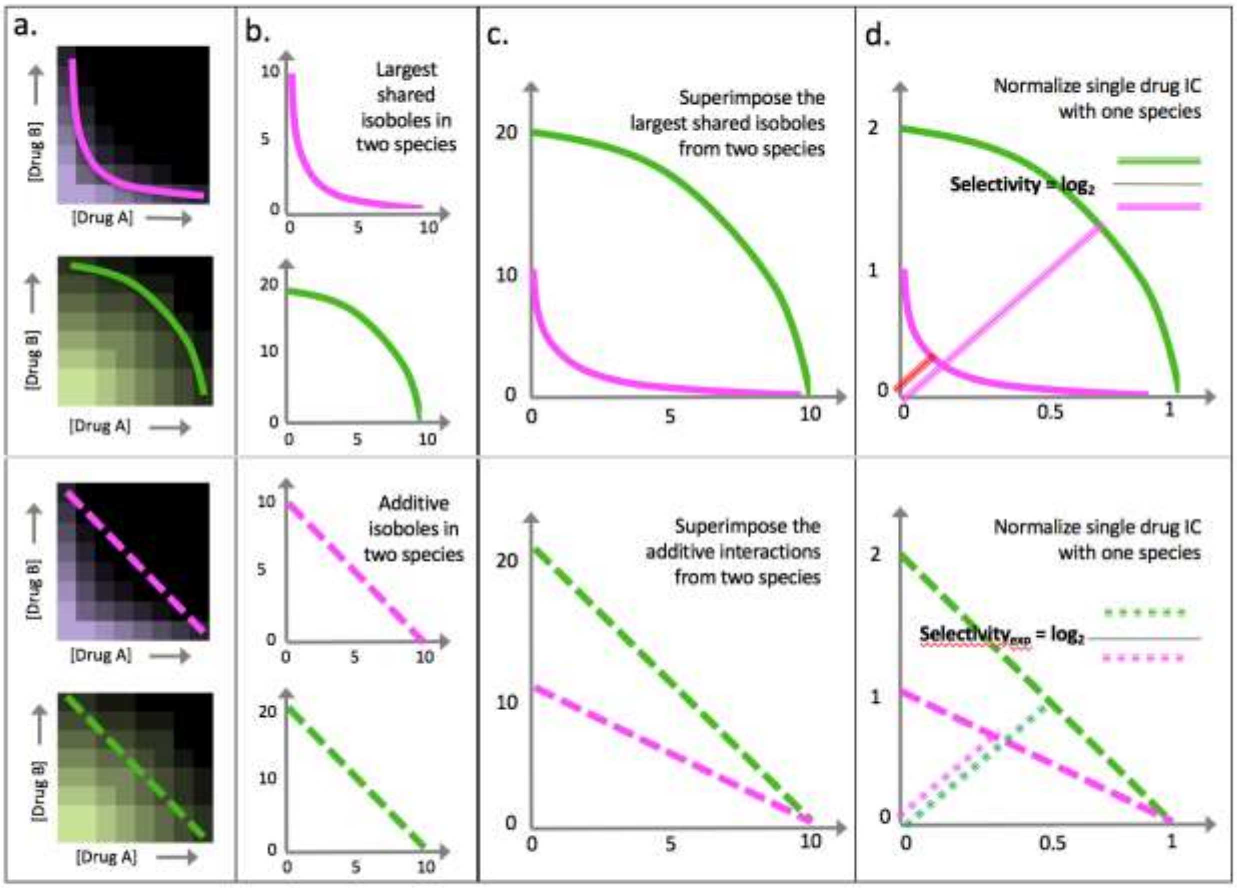

Supplementary Figure 2: Flow diagram of selectivity analysis.

Individual cell types are evaluated for the phenotype of interest in an 8x8 checkerboard assay, wherein two drugs are linearly increased in concentration on each axis (far left panel). The concavity of experimentally derived isoboles (**upper panel**) for both cell types is compared to the null model of Loewe additivity (**lower panel**). For a growth inhibition assay, this corresponds to isoboles connecting regions of similar inhibitory level. The longest isobole for the maximum level of inhibition achieved in both drug interaction assays is selected for further analysis **(a)**. Isoboles for each cell type are superimposed on a dose-response grid, taking into account the concentration of drug required for the given phenotype of interest **(b)**. Drug doses are scaled to one species to allow for multiple comparisons across drug pairs and phenotypes **(c)**. Considering the drug response grid, selectivity is determined as the $\log_2$ of the relative distance from the origin to the isobole. Positive selectivity scores correspond to selection for one species, negative selectivity scores correspond to selection for the other species **(d)**. Selectivity due to drug interactions is further determined by subtracting expected selectivity from observed selectivity.

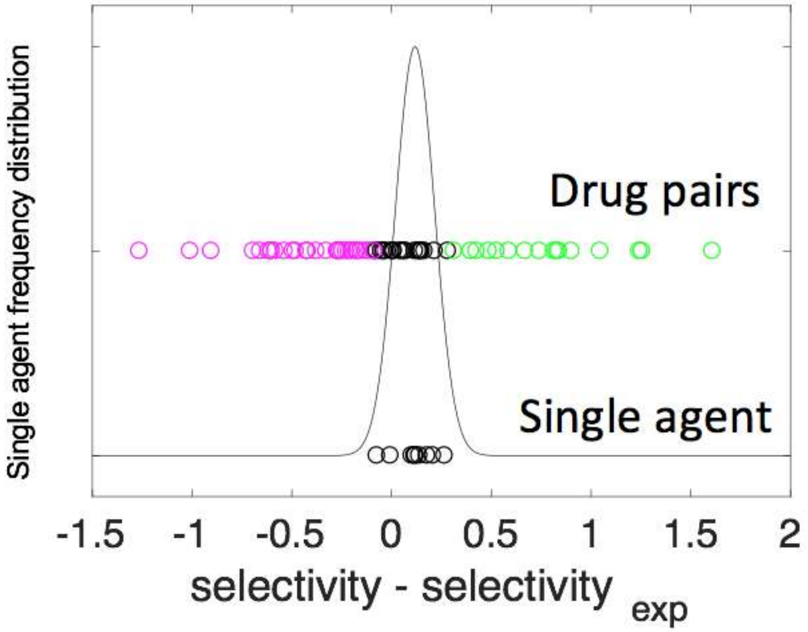

Supplementary Figure 3: Drug combinations have a greater range of selectivity than single agents.

For each drug pair in our study, selectivity – $selectivity_{exp}$ scores were determined for θ = 45 for Supplementary Figures using the equation $\log_2(d_{albicans}/d_{cerevisiae})$. The black circles on the bottom row represent single agent selectivity due to drug interactions, as computed by observed selectivity – $selectivity_{exp}$ values for self-self controls. The difference between observed and expected selectivity for self-self controls are distributed around 0, as expected (range: -0.07 to 0.27). The normal distribution fit to self-self controls is given in black (mean = 0.12, stdev = 0.1). In the top row of circles, selectivity – $selectivity_{exp}$ values for non self-self pairs are given. Drug pairs that significantly select for *C. albicans* (green, positive) or *S. cerevisiae* (magenta, negative) versus additive approximation of expected selectivity were identified based on the 95% confidence interval of the self-self combinations.

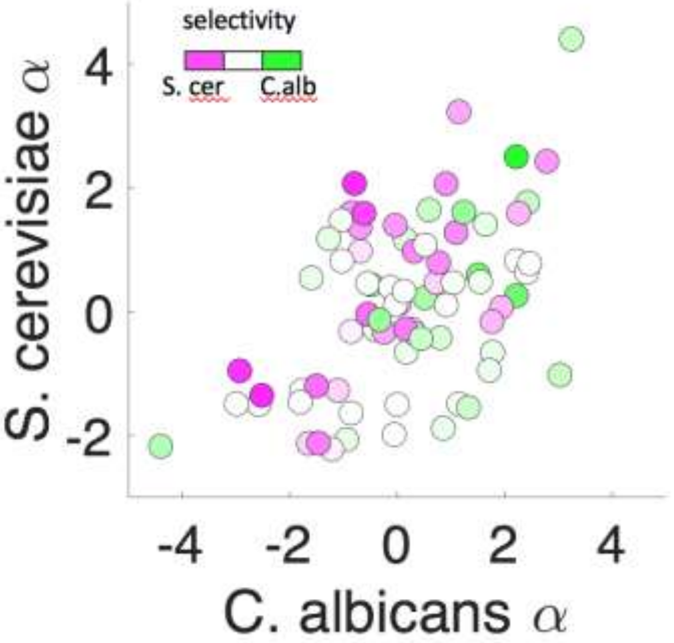

Supplementary Figure 4: Selectivity metric with respect to drug interaction scores (α).

A comparison of α_{cer} versus α_{alb} with overlaid indicators of selectivity (magenta for *S. cerevisiae* selective, green for *C. albicans* selective combinations) demonstrates that drug combinations with surprisingly high selectivity against one species do not always involve a synergistic drug pair. Expected and observed selectivity scores were determined for θ = 45 for Supplementary Figures using the equation $\log_2(d_{albicans}/d_{cerevisiae})$.

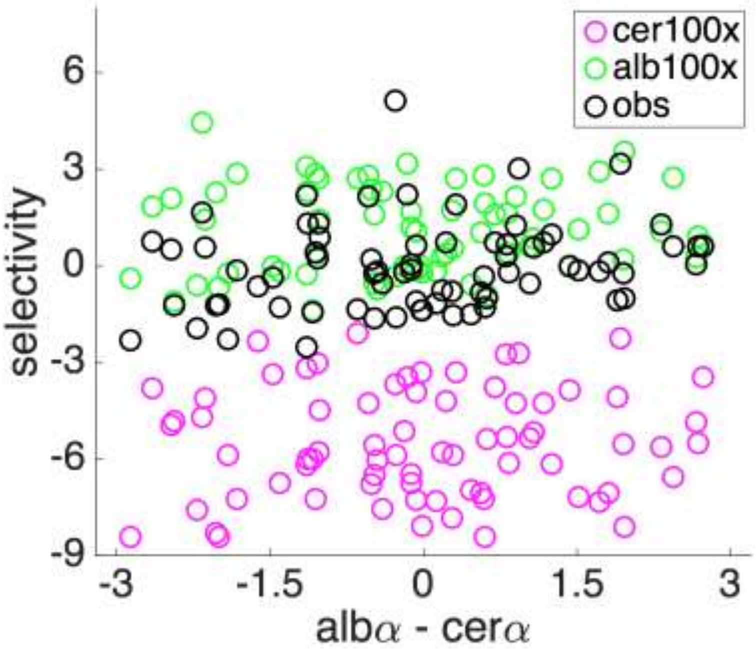

Supplementary Figure 5: Selectivity metrics with respect to delta-α drug interaction scores by level of resistance.

Generally, a large difference in α scores confers a selective advantage to the species with the higher α score given a relatively similar (within 1 order of magnitude) minimal inhibitory concentration for drug pairs tested in each species. There was a weak but significant correlation ($r = 0.26$, $p = 0.02$) between selectivity and the difference of α scores between *C. albicans* and *S. cerevisiae* ($\alpha_{alb} - \alpha_{cer}$) for the tested drug pairs (black circles). In order to understand the influence of antimicrobial resistance on therapeutic selectivity, we modeled the effects of a 100-fold change in minimal inhibitory concentration on selectivity metrics for one drug of each pair in either *S. cerevisiae* (magenta circles) or *C. albicans* (green). We assumed that isophenotypic contours scaled with changes in drug sensitivity (Wood 2014). We found that a large change in MIC overcame the influence of differential drug interactions in our model system and no correlation remained for ($\alpha_{alb} - \alpha_{cer}$) and selectivity scores for simulated resistance in *S. cerevisiae* ($r = 0.09$, $p = .45$) or *C. albicans* ($r = 0.07$, $p = 0.58$). Expected and observed selectivity scores were determined for $\theta = 45$ using the equation $\log_2(d_{albicans}/d_{cerevisiae})$.

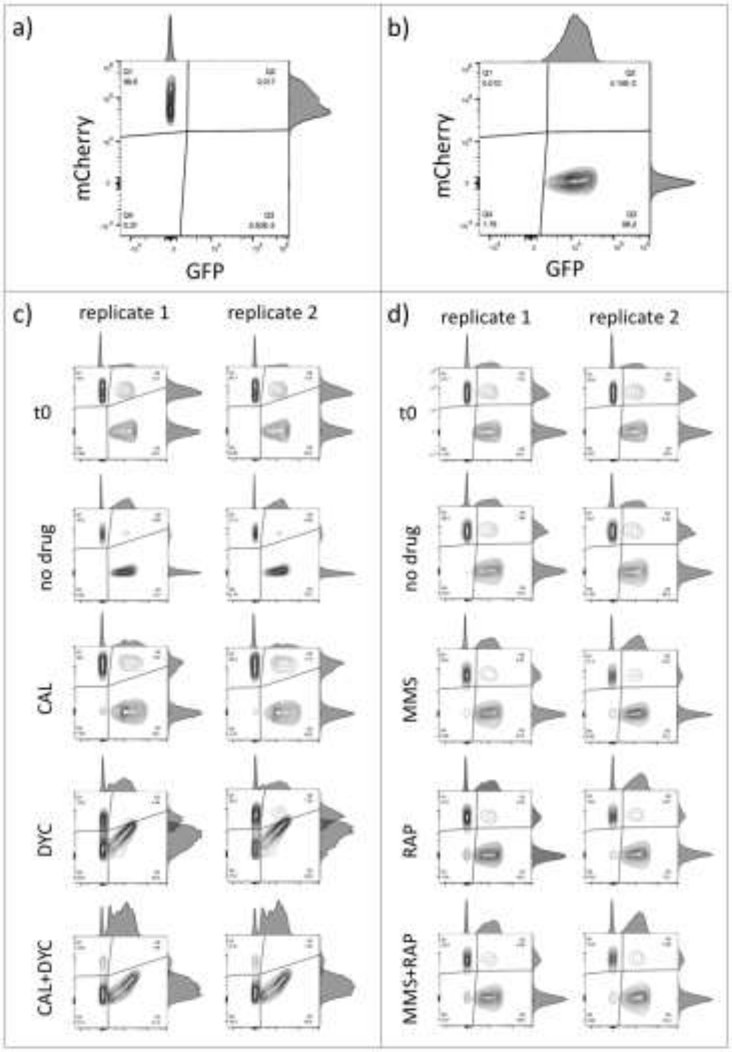

Supplementary Figure 6: Validation of the selectivity model using flow cytometry.
(a) Contour plots of mCherry and GFP expression data for monoculture mCherry+ *S. cerevisiae* (left) and GFP+ *C. albicans* (right), assessed by flow cytometry. The quadrant gates were set up using the pure culture untreated controls to make sure they appropriately separate the two GFP+/mCherry- and GFP-/mCherry+ species. Moreover, they allow for separation, and therefore exclusion, of the GFP-/mCherry- debris or dead cells and GFP+/mCherry+ doublets or double-labeled cells. **(b)** Contour plots of mCherry and GFP expression data for before treatment (t0), no drug, CAL, DYC or CAL+DYC treatments of co-cultures of *S. cerevisiae* and *C. albicans*, for two replicate experiments are shown. Upon DYC treatment, *C. albicans* cells exhibit a shift in fluorescence possibly due to morphological change or autofluorescence. To avoid erroneously classifying Dyc treated *C. albicans* cells as doublets, the quadrant gate was adjusted such that both treated (CAL+DYC, CAL or DYC plots) and untreated (t0, no drug controls) populations are correctly classified. (c) Contour plots of mCherry and GFP expression data for before treatment (t0), no drug, MMS, RAP or MMS+RAP treatments of co-cultures of *S. cerevisiae* and *C. albicans*, for two replicate experiments are shown. In all figures, %of cells in each quadrant are indicated on the contour plots. On the top and right sides of each contour plot, histograms for mCherry and GFP expression are shown, respectively, corresponding to *S. cerevisiae* and *C. albicans* populations. GFP-/mCherry- and GFP+/mCherry+ quadrants are excluded in all histograms and analysis.

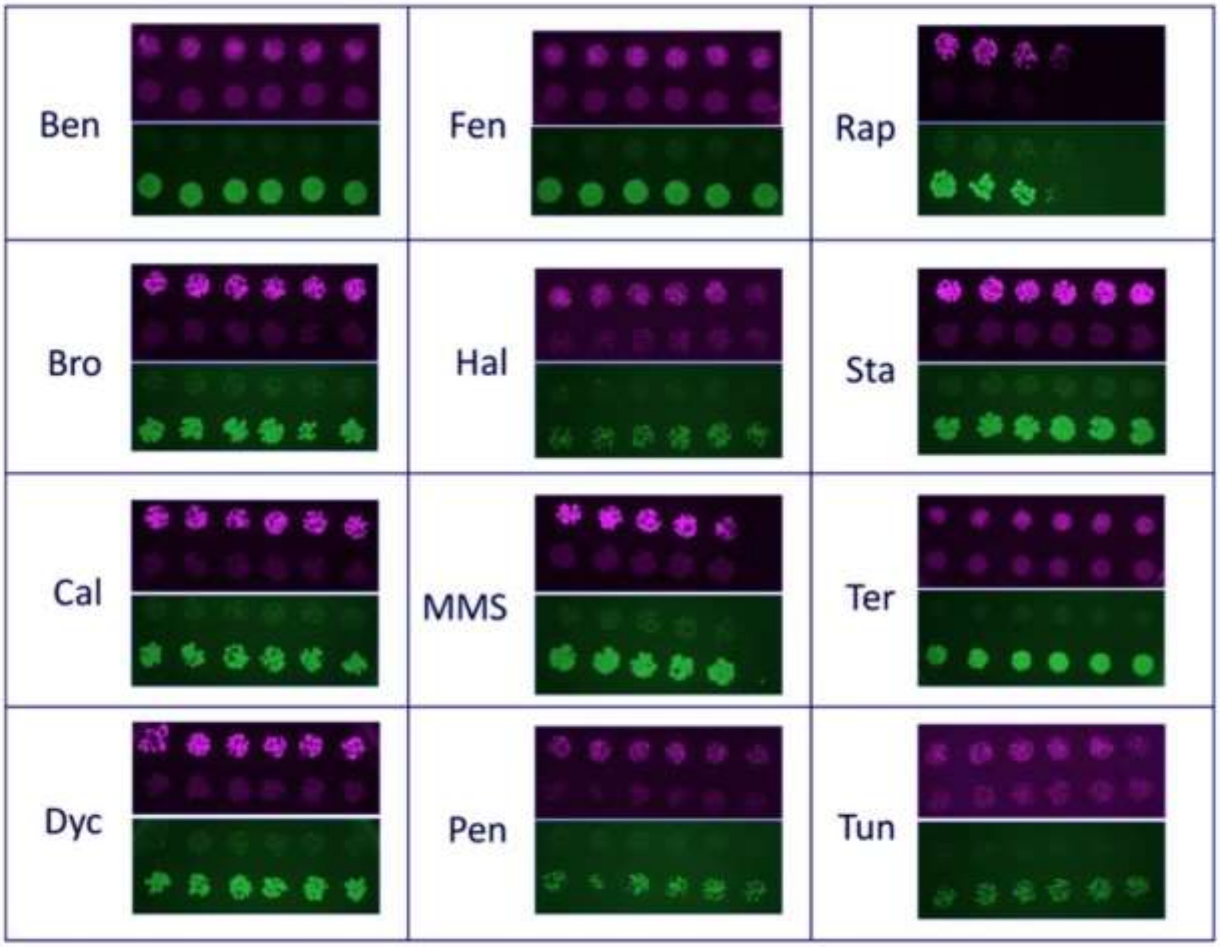

Supplementary Figure 7: Evaluation of fungicidal activity of 12 compounds.

In order to evaluate the suitability of the 12 compounds used in this study for a heterogenous co-culture assay, we tested all 12 agents for fungicidal activity. Shown are dose-response experiments for the fungicidal activity for the corresponding drugs, where *S. cerevisiae* (magenta) and *C. albicans* (green) cells were treated with two-fold increasing dose of drugs and spotted on agar plates on two rows. These two rows of colonies are photographed for mCherry and GFP, to observe *S. cerevisiae* and *C. albicans* colonies, respectively. Only MMS and Rapamycin had rapid fungicidal activity. Therefore, the combination of MMS and Rapamycin was amenable for further illustration of selectivity in an assay for drug combinatorial effects for fungicidality.

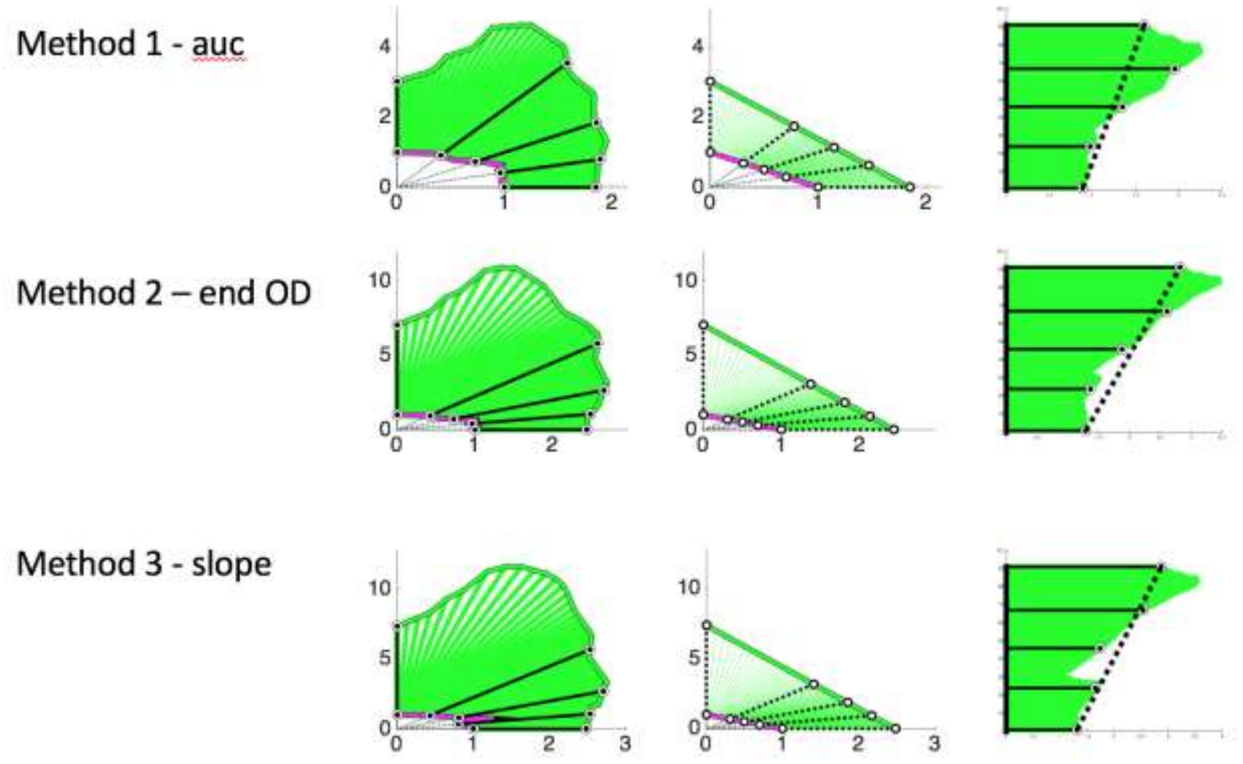

Supplementary Figure 8: Comparison of MMS-Rap selectivity score by growth metrics.

In order to evaluate the influence that growth curve interpretation methods may have on the selectivity of the MMS-Rap drug combination, we reanalyzed all growth metrics for this drug interaction experiments. We found that the relative selectivity due to drug interactions was maintained for this drug pair, though the predicted magnitude of selectivity varied with growth curve interpretation metrics.

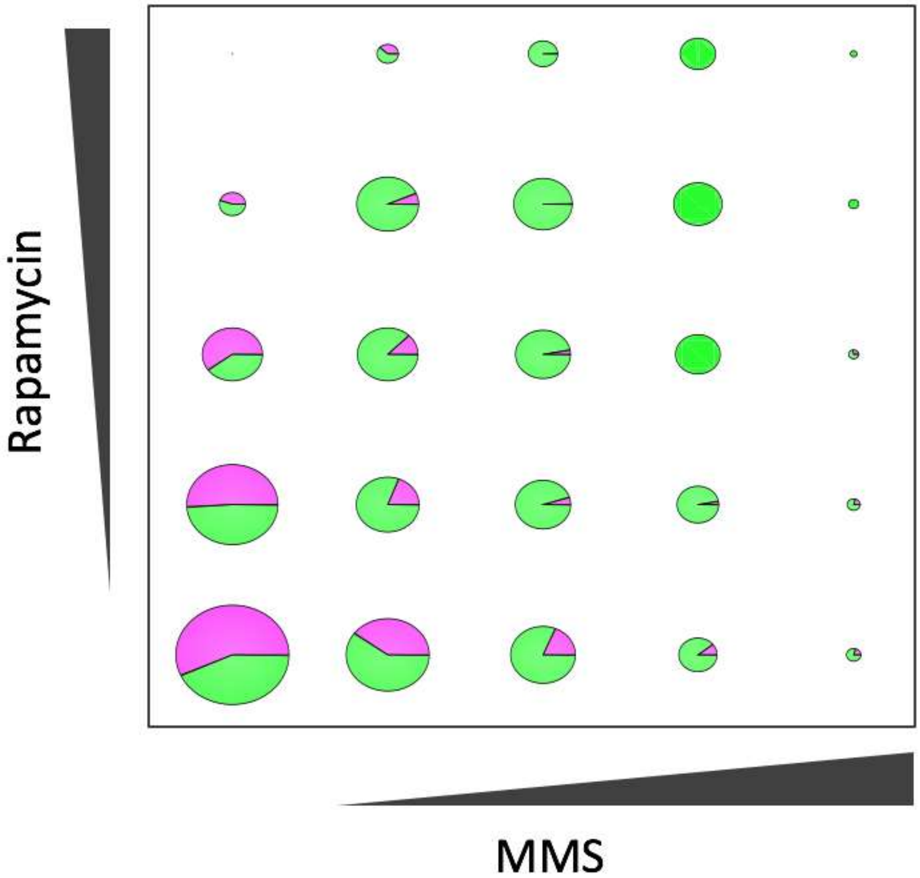

Supplementary Figure 9: Colony counts of MMS-Rap co-culture assay.

In an alternative visualization for our MMS-Rapamycin co-culture assay results, the size of each circle is proportional to the number of total cells that survived a treatment. Green and magenta regions correspond to correspond to *C. albican*s and *S. cerevisiae* colony counts, respectively, showing the relative ratio of two species.

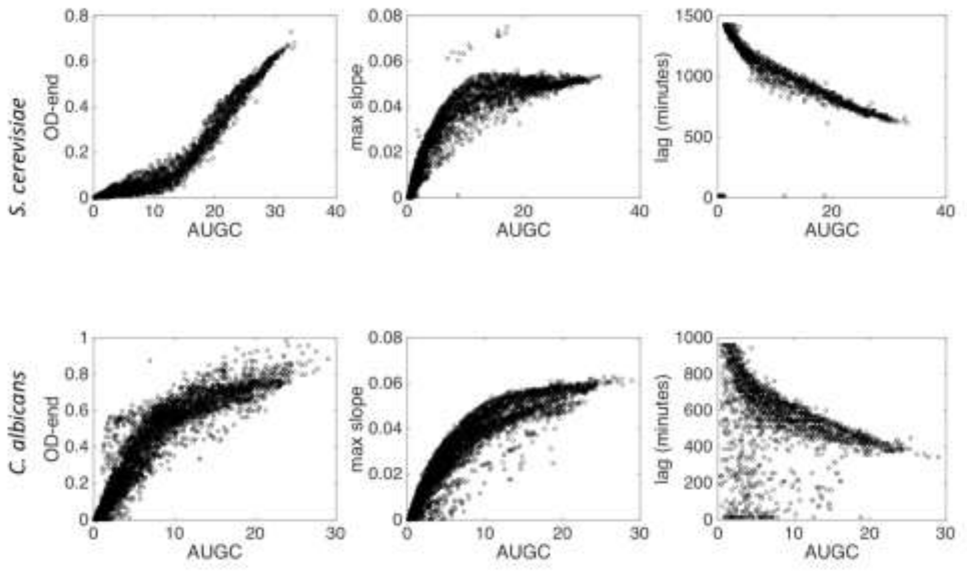

Supplementary Figure 10: Comparison of growth metrics.

In order to evaluate the influence that growth curve interpretation methods may have on growth metrics, we reanalyzed all growth data from drug interaction experiments. As alternatives to area under the growth curve (AUGC), we determined the maximum slope, end-point optical density and lag time for each growth curve. We found that the growth metrics methods were significantly correlated in both *S. cerevisiae* (top panel) and *C. albicans* (bottom panel). AUGC and max slope and end-point OD_{600}, respectively, were significantly positively correlated; r = 0.96 r = 0.95 for *C. albicans*; r = 0.96, r = 0.96 for *S. cerevisiae*). Lag time is given as the recording interval at which the OD surpassed a given threshold (OD_{600} = 0.2). AUGC and lag time were significantly negatively correlated (r = -0.84 for *S. cerevisiae*, r = -0.53 for *C. albicans*).

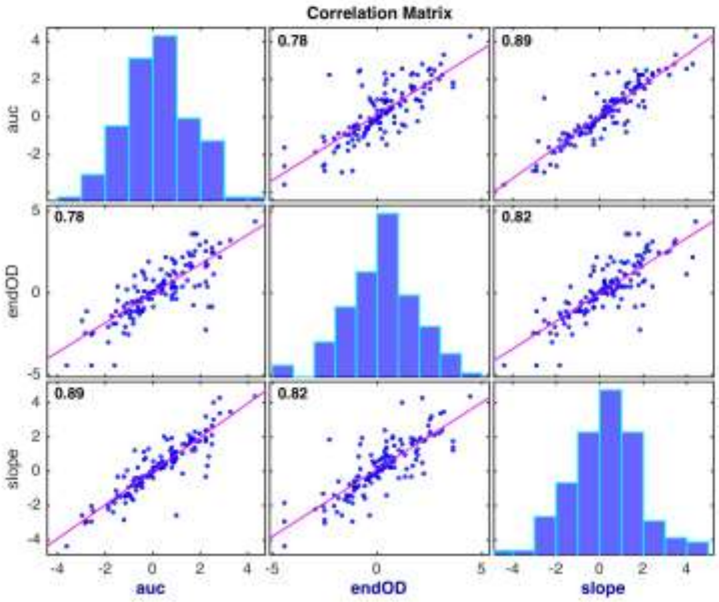

Supplementary Figure 11: Comparison of interaction score by growth metrics.

In order to evaluate the influence that growth curve interpretation methods may have on interaction metrics, we reanalyzed all growth data from drug interaction experiments. We found that interaction scores were significantly correlated for these variable growth metrics. Correlation scores are shown in each subplot.

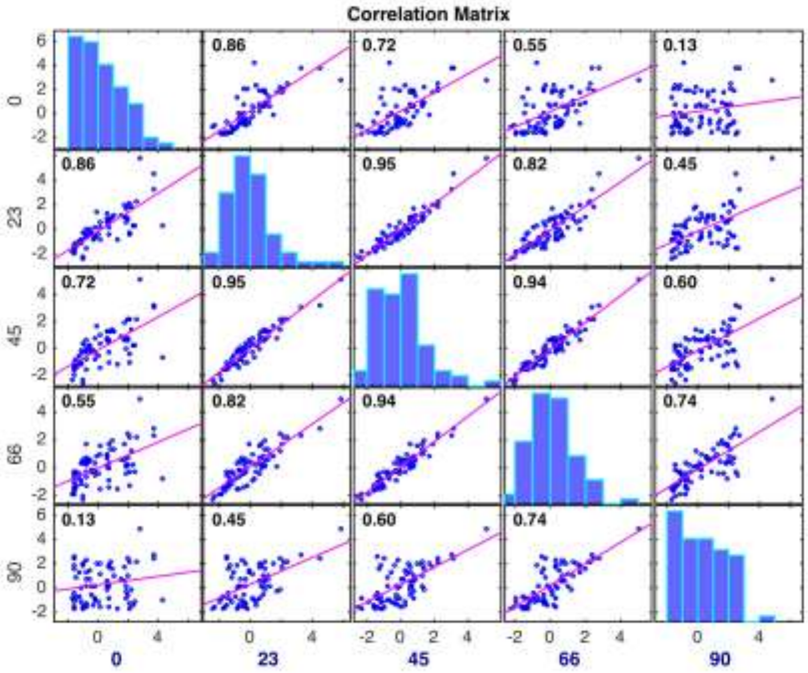

Supplementary Figure 12: Observed selectivity by angle of checkerboard assay.

In order to evaluate how selectivity fluctuates by region of the checkerboard assay, we compared the selectivity scores for the diagonal of the checkerboard (θ = 45) to regions with varying ratios of each drug (θ = 23, θ = 66), as well as to single drug selectivity (θ = 0, θ = 90). Selectivity for θ = 45 was strongly correlated with selectivity at θ = 23 and θ = 66. As expected, single drug selectivity for two drugs, given independently, are uncorrelated.

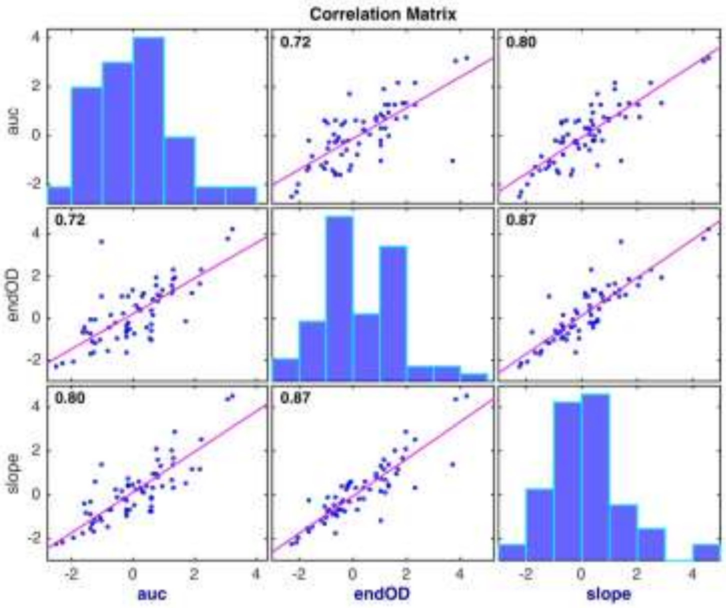

Supplementary Figure 13: Comparison of selectivity score by growth metrics.

In order to evaluate the influence that growth curve interpretation methods may have on selectivity metrics, we reanalyzed all growth data from drug interaction experiments. We found that selectivity scores were significantly correlated for these variable growth metrics. Correlation scores are shown in each subplot.
