# Supplementary material for: Modeling the impact of drug interactions on therapeutic selectivity
Source: Nat Commun. 2018 Aug 27;9:3452. doi: 10.1038/s41467-018-05954-3 (PMC6110842; doi:10.1038/s41467-018-05954-3)
Supplement: Supplementary file 3 — Description of Additional Supplementary Files [file 41467_2018_5954_MOESM3_ESM.pdf]

Description of Additional Supplementary Files

File Name: Supplementary Data 1
Description: Growth measurements used for constructing the selectivity model. (i) Raw cell growth measurements for drug interaction assays of 76 pairwise combinations in *C. albicans* and 8 pairwise combinations in *S. cerevisiae*. Each file contains a 64 column matrix of numbers, which corresponds to OD595 readings for one drug-drug interaction. Rows correspond to different time points with 15 minutes intervals. Columns correspond to the 8 x 8 matrix of drug concentration combinations. (ii) dose-response measurements for 12 drugs in *C. albicans* and *S. cerevisiae*.
